# In Cardiac Patients β-Blockers Attenuate the Decrease in Work Rate during Exercise at a Constant Submaximal Heart Rate

**DOI:** 10.1249/MSS.0000000000003230

**Published:** 2023-05-31

**Authors:** GIOVANNI BALDASSARRE, VALERIA AZZINI, LUCREZIA ZUCCARELLI, CRISTINA DEGANO, FRANCESCO GRANIERO, GLORIA PLETT, MIRCO FLOREANI, STEFANO LAZZER, LUCIO MOS, BRUNO GRASSI

**Affiliations:** 1 Department of Medicine, University of Udine, Udine, ITALY; 2 Department of Cardiology, San Daniele del Friuli Hospital, San Daniele del Friuli, ITALY; 3 Physical Exercise Prescription Center, Gemona del Friuli Hospital, Gemona del Friuli, ITALY; 4 School of Sport Sciences, University of Udine, Udine, ITALY

**Keywords:** EXERCISE PRESCRIPTION, EXERCISE TOLERANCE, CARDIOVASCULAR DISEASE, HR KINETICS, β-BLOCKERS

## Abstract

**Purpose:**

Exercise prescription based on fixed heart rate (HR) values is not associated with a specific work rate (WR) during prolonged exercise. This phenomenon has never been evaluated in cardiac patients and might be associated with a slow component of HR kinetics and β-adrenergic activity. The aims were to quantify, in cardiac patients, the WR decrease at a fixed HR and to test if it would be attenuated by β-blockers.

**Methods:**

Seventeen patients with coronary artery disease in stable conditions (69 ± 9 yr) were divided into two groups according to the presence (BB) or absence (no-BB) of a therapy with β-blockers, and performed on a cycle ergometer: an incremental exercise (INCR) and a 15-min “HR_CLAMPED_” exercise, in which WR was continuously adjusted to maintain a constant HR, corresponding to the gas exchange threshold +15%. HR was determined by the ECG signal, and pulmonary gas exchange was assessed breath-by-breath.

**Results:**

During INCR, HR_peak_ was lower in BB versus no-BB (*P* < 0.05), whereas no differences were observed for other variables. During HR_CLAMPED_, the decrease in WR needed to maintain HR constant was less pronounced in BB versus no-BB (−16% ± 10% vs −27 ± 10, *P* = 0.04) and was accompanied by a decreased V̇O_2_ only in no-BB (−13% ± 6%, *P* < 0.001).

**Conclusions:**

The decrease in WR during a 15-min exercise at a fixed HR (slightly higher than that at gas exchange threshold) was attenuated in BB, suggesting a potential role by β-adrenergic stimulation. The phenomenon may represent, also in this population, a sign of impaired exercise tolerance and interferes with aerobic exercise prescription.

Compelling evidence indicates that physical inactivity is implicated in the etiology of numerous chronic diseases that impact negatively on the health status (1,2). On the contrary, regular physical activity improves the quality of life by increasing exercise tolerance and by reducing the risk of all-cause mortality in a dose–response manner (3). Although some physical activity is better than none, an individually tailored exercise prescription is more effective in improving the subjects’ physical performance and eventually their health (4,5). Exercise prescription is often done in terms of exercise domains (4,5), which have distinct characteristics in terms of metabolic responses and fatigue. Too often, however, intensity prescription for aerobic exercise, both in healthy and diseased populations, is defined in terms of work rates corresponding to a fixed percentage of heart rate (HR) reserve or of peak HR (4,6–10), mainly for the facility of tracking HR by wearable HR meters or cell phones. Studies by our group, however, demonstrated that both in healthy young subjects (11,12) and in obese patients (13), a disproportionate increase in HR (“slow component” of HR kinetics) is present during constant work rate exercises. The slow component of HR kinetics occurs at lower work rates (below the gas exchange threshold (GET)) compared with the slow component of pulmonary O_2_ uptake (V̇O_2_) kinetics (11,12). Furthermore, above GET, the relative amplitude of the HR slow component is more pronounced than that of $\dot{V}$O_2_ slow component (11,12). As a consequence of this phenomenon, during exercise carried out for 15 min at a constant HR, slightly above that corresponding to GET, an intensity often recommended by guidelines for aerobic exercise (4,5,14), work rate had to significantly decrease to maintain HR constant, both in healthy young subjects (11,12) and in obese patients (13). At times, the decreased work rate led to changes in exercise domain, from the heavy to the moderate intensity (12). All these obviously make exercise prescription and evaluation based on specific percentages of HR peak potentially inaccurate.

The phenomena described previously have never been investigated in cardiac patients, a population in which exercise prescription is often performed in terms of fixed percentages of HR max (10,15). Therefore, the main aim of the present study was to identify and quantify in coronary artery disease patients in stable conditions the decrease in work rate during exercise performed at a fixed HR, set at a value slightly higher than that corresponding to GET. Considering that this phenomenon represents a biomarker of exercise tolerance (12,13), if confirmed, the proposed approach may be of interest also for diseased populations. We also intended to test the hypothesis that the decrease in work rate, being associated with a slow component of HR kinetics, would be significantly attenuated by the administration of β-blockers (BB). These drugs significantly improve the prognosis of these patients (16) by reducing the β-adrenergic drive, which has been proposed as a potential cause of the HR slow component (13,17).

## MATERIALS AND METHODS

### Patients

Nineteen patients (17 men and 2 women) followed by the Physical Exercise Prescription Center, Department of Medicine, University of Udine, situated in the Hospital of Gemona del Friuli, were selected for this study. All subjects had cardiovascular diseases (17 coronary artery disease, 1 heart failure, and 1 hypertensive cardiac disease) and were in stable clinical conditions. Participants were divided into two groups according to the presence or absence of an ongoing therapy with β1-selective blockers: in group BB (*n* = 10; mean ± SD: age, 69 ± 7 yr; height, 1.75 ± 0.11 m; body mass, 86.0 ± 13.7 kg), patients were treated with BB (atenolol 50 mg·d^−1^ (*n* = 1), or bisoprolol 2.5–7.5 mg·d^−1^ (*n* = 8), or metoprolol 2 × 50 mg·d^−1^ (*n* = 1)), whereas in group no-BB (*n* = 9; mean ± SD: age, 71 ± 11 yr; height, 1.73 ± 0.08 m; body mass, 79.5 ± 11.8 kg), patients were not treated with BB. Other cardiovascular and metabolic medications taken by patients during the study period are also reported in Table 1.

**TABLE 1:** Main cardiovascular and metabolic medications, and relative number of patients (treated with BB or without BB (no-BB)) taking the reported drugs during the study period.

Medications	BB	No-BB
Antiarrhythmics	0	3
Anticoagulants and antiplatelets	8	8
Antidiabetics	2	1
Antihypertensives	8	7
Antihyperuricemic agents	5	1
Anti-ischemic agents	2	1
Diuretics	3	1
Lipid-lowering agents	9	8

One patient (group no-BB) was excluded from the study because of the occurrence of frequent ventricular ectopic beats during the incremental exercise, which led to the premature interruption of the test. Another patient of group BB complained of increasing exertional dyspnea and was excluded because of a moderate anemia ([Hb] 9.6 g·dL^−1^). Therefore, only 17 patients (9 for group BB and 8 for group no-BB) completed the testing procedures and were included in the statistical analysis.

All subjects gave their written informed consent after they received a detailed explanation of the experimental procedures before the start of the study, whose protocol was approved (Prot. IRB: 84/2022; June 6, 2022) by the Institutional Review Board of the Department of Medicine, University of Udine.

### Experimental protocol

Participants were required to come to the laboratory on three separate occasions. On their first visit, subjects underwent a physical examination and anthropometric measurements were performed. During the second visit, the participants completed an incremental exercise on an electronically braked cycle ergometer (Ergoselect 100; Ergoline GmbH) until voluntary exhaustion. Pedaling frequency was digitally displayed to the subjects, who were asked to keep a constant cadence throughout the tests between 60 and 65 rpm. Voluntary exhaustion was defined as the incapacity to maintain the imposed load and pedaling frequency despite vigorous encouragement by the researchers. The incremental exercise protocol consisted of ramp increases of 15–30 W·min^−1^ (preceded by a resting baseline and by 2 min at 10–20 W), depending on the characteristics and the predicted functional capacity of each patient; the aim was to reach voluntary exhaustion in 10–15 min.

During the third visit to the laboratory, patients performed an “HR-controlled” exercise (HR_CLAMPED_), in which work rate was continuously adjusted to maintain a constant HR, equivalent to GET +15%. During the first 2 min of HR_CLAMPED_, work rate was progressively increased to reach the work rate target value; thereafter, it was kept constant for 3 min, and then it was adjusted by the operator by decreasing/increasing by 2 W every 5 s, to maintain HR constant for the remaining 15 min of the exercise.

### Measurements

Pulmonary ventilation (V̇E), O_2_ uptake (V̇O_2_), and CO_2_ output (V̇CO_2_) were assessed breath-by-breath by a metabolic cart (Quark PFTergo; Cosmed, Rome, Italy). Expiratory flow measurements were performed by a turbine flow meter, calibrated before each experiment by a 3-L syringe at three different flow rates. Calibration of O_2_ and CO_2_ analyzers was performed before each experiment by utilizing gas mixtures of known composition. Gas exchange ratio (R) was calculated as V̇CO_2_/V̇O_2_. GET was determined using the “V-slope” method and “secondary criteria” (18); the respiratory compensation point (RCP) was determined by standard criteria (19). To identify the work rate and HR corresponding to V̇O_2_ at GET, the effect of the delayed V̇O_2_ adjustment to the increased work rate during the incremental test was corrected by shifting the linear V̇O_2_ versus time relationship to the left, by an amount corresponding to the mean response time of the V̇O_2_ kinetics (20) of a similar patients’ population (21,22). HR was determined from a 12-lead electrocardiography (Quark C12x; Cosmed).

Mean values of ventilatory and pulmonary gas exchange, and cardiovascular variables were calculated during the last 20 s of each minute of exercise for both the incremental and the HR_CLAMPED_ exercises; values obtained during the exhausting work rate of the incremental exercise were considered peak values.

### Statistical analysis

Results are expressed as mean ± SD values. Statistical significance of the differences between the two groups (BB vs no-BB) was checked by two-tailed Student’s unpaired *t*-tests. Respiratory and cardiovascular variables measured over several time periods during HR_CLAMPED_ exercises were analyzed using a two-way (condition–time) repeated-measures ANOVA. Significant interaction effects were followed up by Tukey *post-hoc* test. Single and two linear regressions were carried out by the least-squared residuals method. Comparisons between the two fitting models were carried out using the *F*-test. The level of significance was set at 0.05. Statistical analyses were carried out by a commercially available software package (Prism 8.0; GraphPad).

## RESULTS

Peak values of the main respiratory and cardiovascular variables obtained during the incremental exercises are reported in Table 2. No significant differences were found in BB versus no-BB for V˙O_2peak_, peak work rate, and other variables ( $\dot{V}C$O_2_, R, $\dot{V}E$, tidal volume, breathing frequency, end-tidal O_2_ partial pressure, end-tidal CO_2_ partial pressure) determined at peak exercise. As expected, HR_peak_ was significantly lower in BB than in no-BB, both when the variable was expressed in breaths per minute or in percentage of the age-predicted maximum values (calculated as 208 − 0.7 · age [23]). More specifically, expressed as a percent of the age-predicted maximum, HR_peak_ values were about 90% in no-BB and about 70% in BB, and the extent of the HR_peak_ decrease after BB administration (about −20%) was similar to that reported in the literature (24).

**TABLE 2:** Peak values of the main respiratory, cardiovascular, and metabolic variables determined during the incremental exercise in coronary artery disease patients in stable conditions treated with BB or not treated with BB (no-BB).

	BB	No-BB	*P*
Peak work rate (W)	155 ± 50	165 ± 55	0.71
V̇O_2peak_ (L·min^−1^)	1.814 ± 0.501	1.753 ± 0.510	0.81
V̇O_2peak_ (mL·kg^−1^·min^−1^)	21.2 ± 4.8	22.9 ± 8.5	0.61
V̇CO_2peak_ (L·min^−1^)	2.050 ± 0.587	2.180 ± 0.652	0.67
R_peak_	1.13 ± 0.10	1.24 ± 0.13	0.06
V̇E_peak_ (L·min^−1^)	80.5 ± 20.1	77.3 ± 21.7	0.76
V_Tpeak_ (L)	2.22 ± 0.51	2.06 ± 0.46	0.50
fR_peak_ (breaths per minute)	37 ± 6	37 ± 6	0.73
PetO_2peak_ (mm Hg)	118.1 ± 3.6	116.9 ± 5.5	0.60
PetCO_2peak_ (mm Hg)	31.4 ± 4.0	33.9 ± 4.7	0.26
HR_peak_ (bpm)	114 ± 19*	139 ± 23	0.03
HR_peak_ (%HR_max pred_)	71 ± 12*	88 ± 11	0.01
V̇O_2GET_ (L·min^−1^)	1.198 ± 0.211	1.150 ± 0.285	0.70
V̇O_2GET_ (%V̇O_2peak_)	67 ± 12	66 ± 6	0.82
Work rate_GET_ (W)	62 ± 18	62 ± 28	0.93
HR_GET_ (bpm)	81 ± 9	90 ± 17	0.18
V̇O_2RCP_ (L·min^−1^)	1.603 ± 0.405	1.566 ± 0.418	0.86
V̇O_2RCP_ (%V̇O_2peak_)	89 ± 6	90 ± 5	0.83

Data are means ± SD. *P* values relate to differences between groups by means of Student’s unpaired *t*-test. Data related to the GET and to the RCP are also presented.

fR, breathing frequency; HR_GET_, HR at GET; HR_max pred_, age-predicted maximal HR; PetCO_2_, end-tidal CO_2_ partial pressure; PetO_2_, end-tidal O_2_ partial pressure; V̇CO_2peak_, CO_2_output; V̇E_peak_, pulmonary ventilation; V̇O_2GET_, pulmonary oxygen uptake at GET; V̇O_2peak_, pulmonary oxygen uptake; V̇O_2RCP_, pulmonary oxygen uptake at RCP; V_T_, tidal volume; Work rate_GET_, work rate at GET.

V˙O_2_, work rate, and HR at GET, as well as V˙O_2_ at RCP were not different between the two groups. Both in BB and no-BB, $\dot{V}$O_2_ at GET and at RCP corresponded to about 65% and about 90% of V˙O_2peak_, respectively.

**FIGURE 1:**
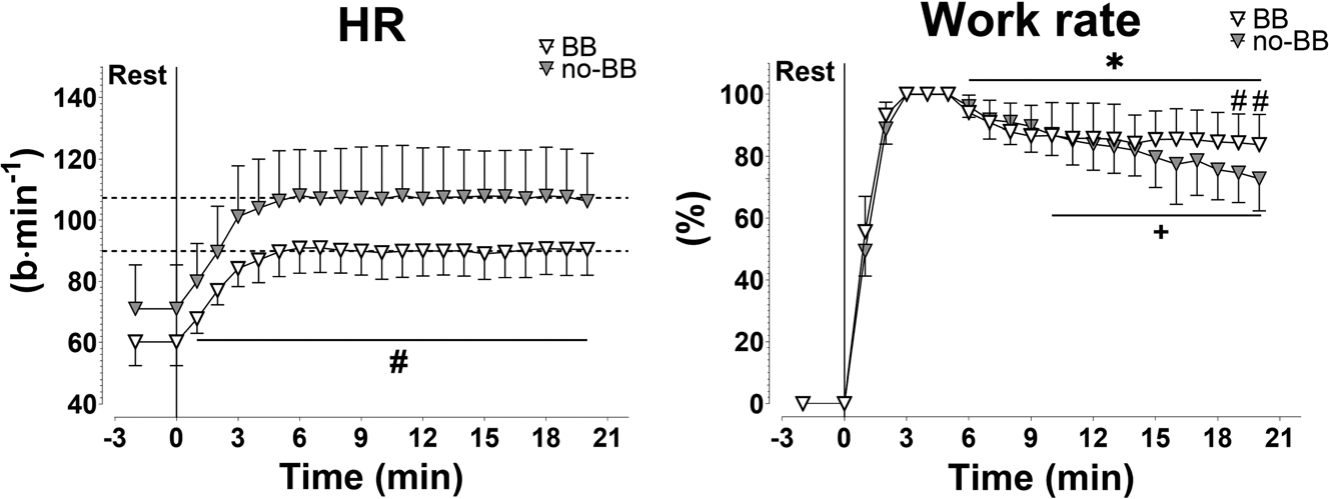
Mean ± SD values of HR and work rate during HR_CLAMPED_ exercises in patients treated with BB and in patients not treated with BB (no-BB). The *horizontal dashed line* indicates the mean HR target value. *,+Statistically different from the highest value of the variable (*P* < 0.05). #Statistically different from the value obtained in the no-BB group (*P* < 0.05). See text for further details.

In Figure 1, mean ± SD values of HR and work rate obtained during the HR_CLAMPED_ exercise are shown. In both groups, HR mean target value (set at 90 ± 8 and 107 ± 16 beats per minute in BB and no-BB, respectively, corresponding to 115% of GET) was reached within the first 5 min of exercise and remained constant throughout the test, indicating that the aim of the protocol (keeping HR constant) was successfully reached in both groups. HR values during exercise were significantly lower in BB than in no-BB. In both groups, work rate had to decrease to keep HR constant, and the decrease was more pronounced in no-BB versus BB.

**FIGURE 2:**
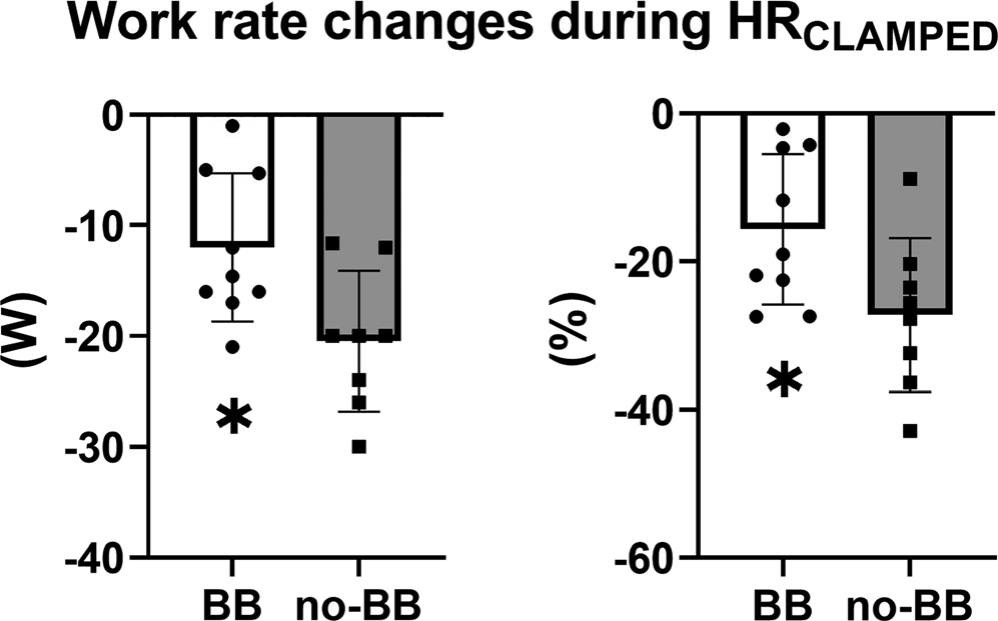
Individual and mean ± SD values of the absolute and percentage changes in work rate during the HR_CLAMPED_ exercise in cardiac patients treated with BB or not treated with BB (no-BB). *Statistically different from the value obtained in the no-BB group (*P* < 0.05).

The different work rate decreases in the two groups are more clearly evident in Figure 2, in which individual and mean ± SD values of the decreases of the variable from the 3rd to 20th minutes of exercise are presented. Work rate decreases in no-BB were significantly greater than in BB, both when expressed in W (−12 ± 7 vs −20 ± 6 W, *P* = 0.02; left panel) and as a percent of the third minute value (−16% ± 10% vs −27% ± 10%, *P* = 0.04; right panel).

**FIGURE 3:**
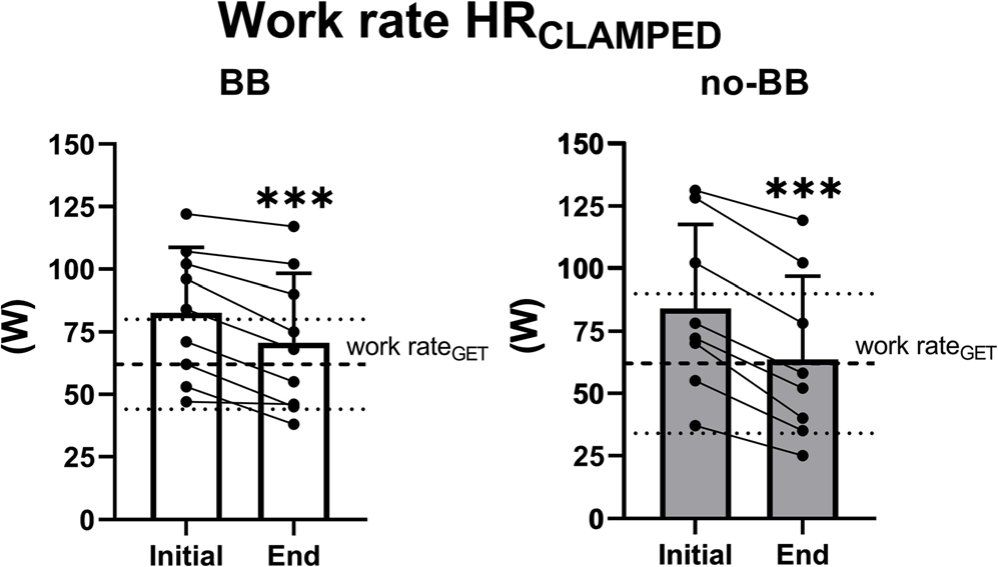
Individual and mean ± SD values of work rate at the 5th (Initial) and 20th minutes (End) of the HR_CLAMPED_ exercise in cardiac patients treated with BB or not treated with BB (no-BB). The *horizontal dashed line* indicates the mean value of the work rate at the GET, whereas the *two horizontal dotted lines* indicate its SD. ***P < 0.001. See text for further details.

Interestingly, the work rate decreases determined a shift in the exercise intensity domains (Fig. 3). Both in BB and no-BB, the mean values of work rate were above GET (i.e., in the heavy-intensity domain) at the third minute of exercise, but were significantly reduced as exercise progressed to maintain HR constant, becoming similar to GET (i.e., at the boundary between moderate-intensity and heavy-intensity domains) at the end of the exercise. Individual values in no-BB (left panels) show that at the end of the exercise, five of 8 patients were exercising below GET, that is, in the moderate-intensity domain.

**FIGURE 4:**
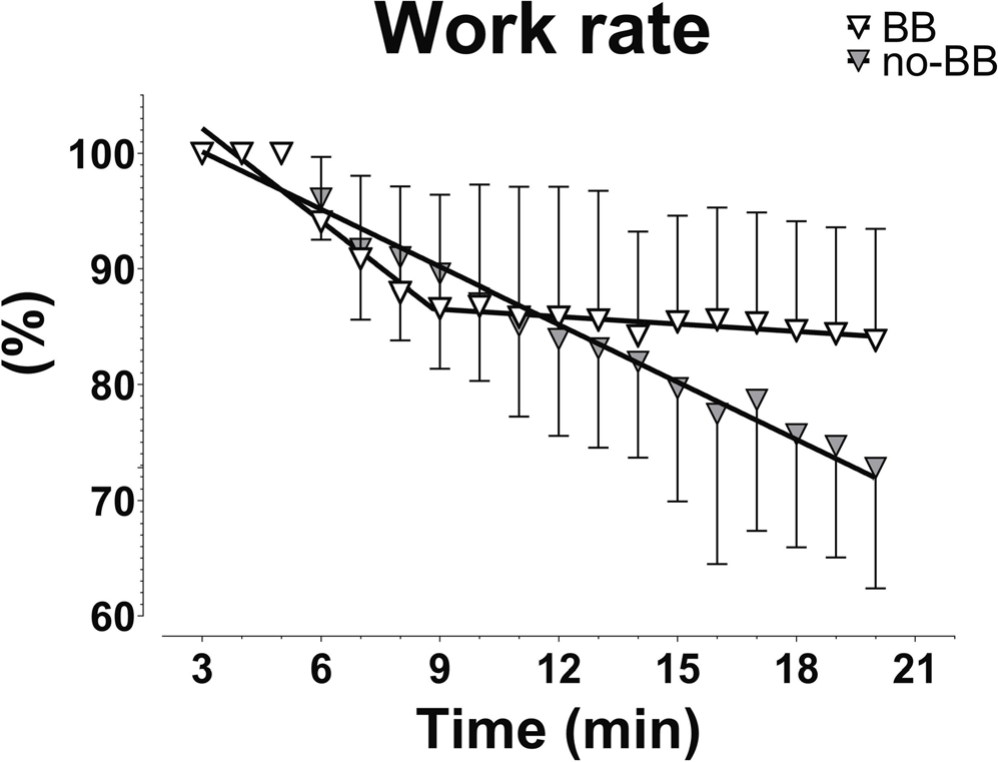
Mean ± SD values of work rate from the 3rd to 20th minutes of HR_CLAMPED_ exercises in patients treated with BB and in patients not treated with BB (no-BB). The fitted single and two linear regression lines are also shown. See text for further details.

Also, the time courses of the work rate decreases in the two groups seem to be of interest. In Figure 4, the work rate data presented in the left panel of Figure 1 are shown with expanded *x* (from minute 3 to minute 20) and *y* axes, to better appreciate the time courses of the variables. Whereas, in no-BB, the work rate decrease versus time followed a linear function throughout the period, in BB, after a linear decrease from minute 3 to about minute 9 (this linear decrease was substantially superimposed on that observed in no-BB), the variable kept substantially constant until the end of the exercise.

The equation that best fitted the experimental data (single vs two linear regressions) was determined using the *F*-test (see Statistical analysis). Implications of these different time courses will be analyzed in the Discussion section.

**FIGURE 5:**
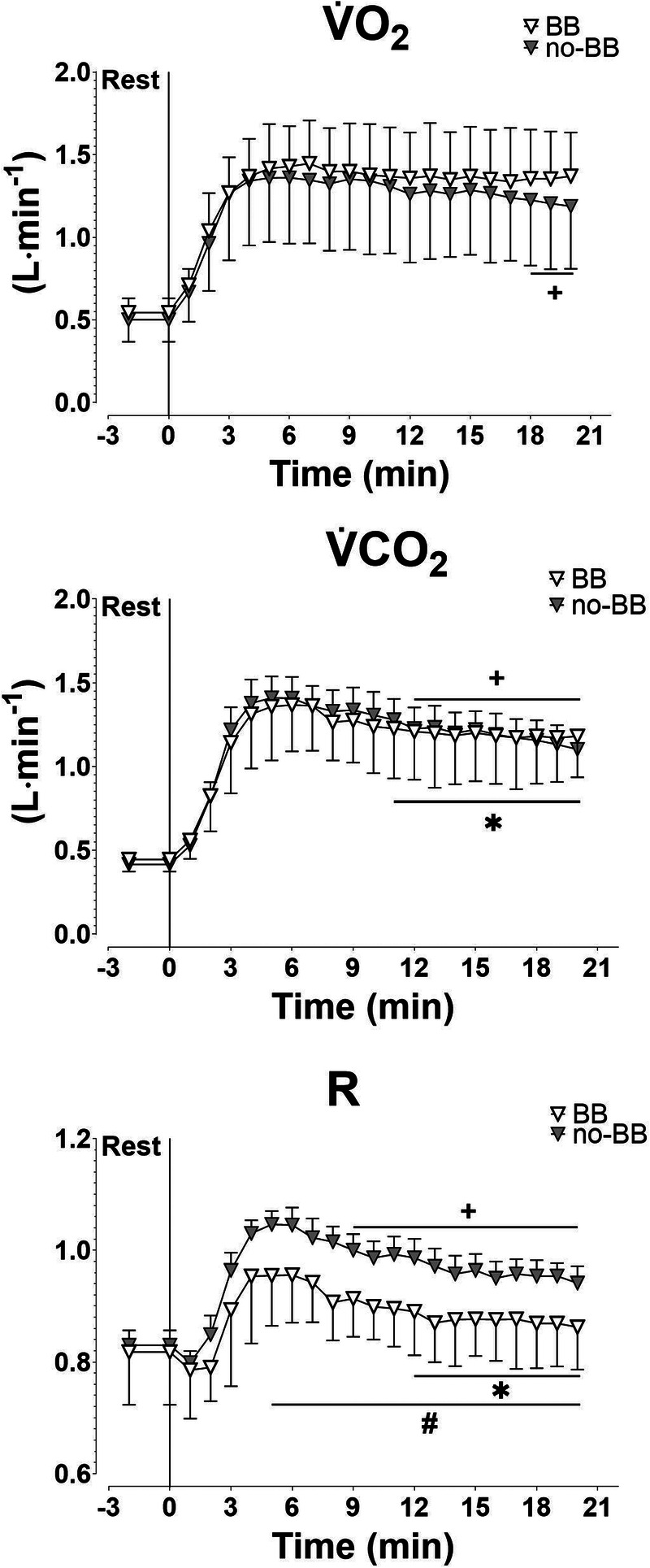
V̇O_2_, V̇O_2_, and R during HR_CLAMPED_ exercises in patients treated with BB and in patients not treated with BB (no-BB). *,+Statistically different from the highest value of the variable (*P* < 0.05). #Statistically different from the value obtained in the no-BB group (*P* < 0.05). See text for further details.

**FIGURE 6:**
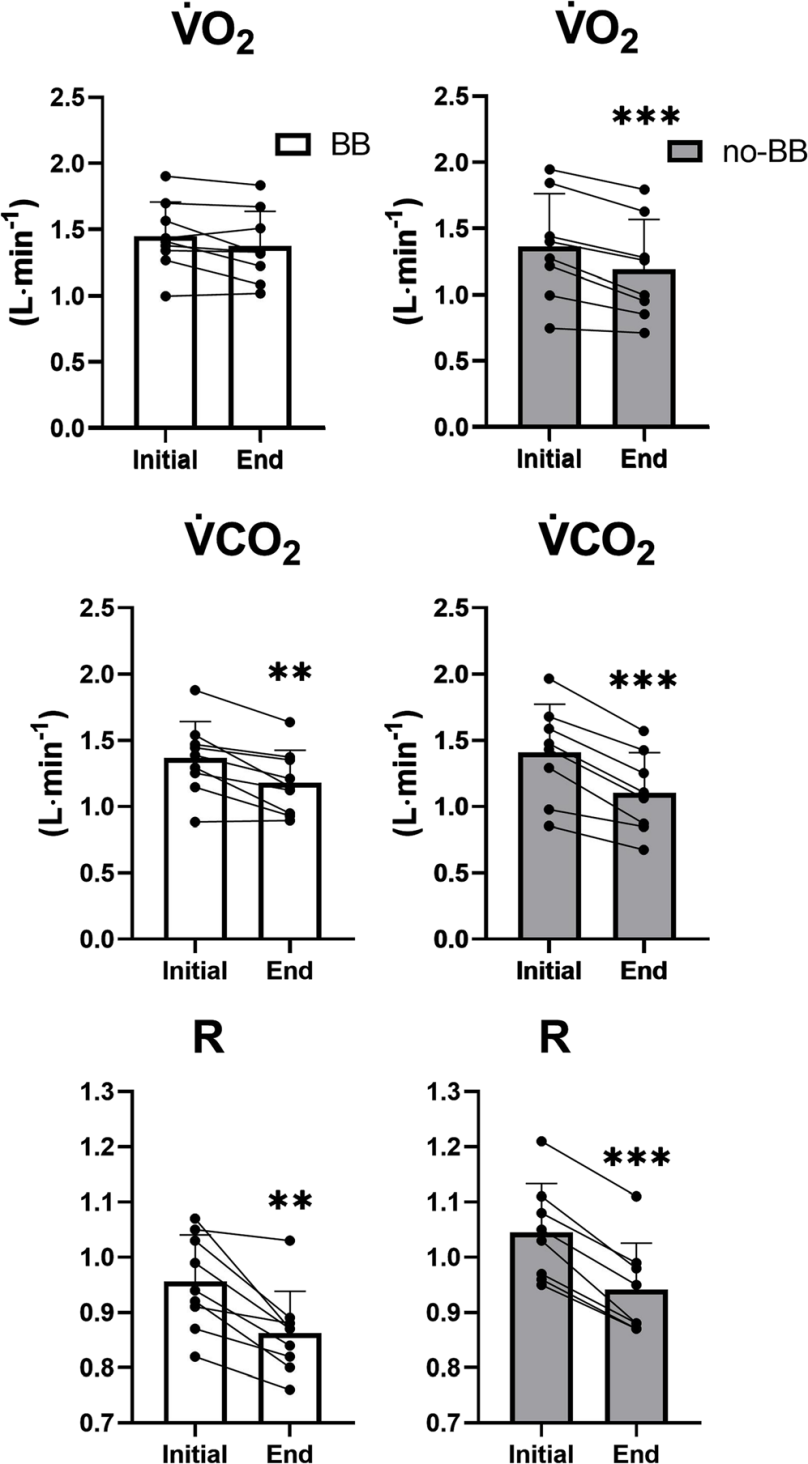
Individual and mean ± SD highest (Initial) and last minute (End) values of V̇O_2_, V̇CO_2_, and R recorded during HR_CLAMPED_ exercise in cardiac patients treated with BB and in patients not treated with BB (no-BB). **P < 0.01; ***P < 0.001.

Mean ± SD values of V̇O_2_, V̇CO_2_, and R obtained during the HR_CLAMPED_ exercise are shown in Figure 5. Individual and mean ± SD values of the of the variable at the 3rd and 20th minutes of exercise are presented in Figure 6. V̇O_2_ did not change in BB, whereas it significantly decreased in no-BB. V̇CO_2_ and R decreased in both groups.

## DISCUSSION

During a 15-min exercise set at a work rate corresponding to an HR slightly above GET, patients with coronary artery disease in stable conditions have to significantly decrease work rate to keep HR constant. Confirming our hypothesis, the work rate decrease at a fixed HR was attenuated by BB administration, suggesting a potential role for the phenomenon attributable to β-adrenergic stimulation. The decreased work rate at a fixed HR slightly above GET, observed in the present study in cardiac patients, confirms previous observations by our group in young subjects (11), obese adolescents (13), and young subjects undergoing bed rest (12).

Textbook of physiology says that a higher HR for the same work rate indicates a decreased exercise tolerance (25–27). In the present study and in our previous ones (11–13), we observed a “mirror image” of the phenomenon, that is, a lower work rate for the same HR. We postulate that the observation represents a sign, or a biomarker, of impaired exercise tolerance as well. In support of this concept are the observations that the work rate decrease at a fixed HR was smaller after exercise training (13) and was greater after bed-rest deconditioning (12). In the present study, we demonstrate that this biomarker of impaired exercise tolerance can be identified also in patients with cardiovascular diseases. The approach should be of interest also from a practical point of view, because it is based on variables (HR and work rate), which can be easily determined with great precision during exercise, carried out with different ergometers or even in field studies. Moreover, the method does not need gas exchange measurements, or the need for the subject/patient to perform several repetitions of submaximal, maximal, and supramaximal exercises, as imposed by other approaches frequently utilized to evaluate exercise tolerance, and based on the determination of the power–duration curve and critical power (28). If adequately standardized, the approach described in the present study may be valuable also in diseased populations. The observed phenomena (decreases in work rate and (in no-BB) in V̇O_2_ at a fixed HR) represent a sort of a mirror image of the progressive increases in HR and V̇O_2_ as a function of time (traditionally termed “slow component”) observed during heavy-intensity (above GET) constant work rate exercise (11–13,29,30). Whereas the V̇O_2_ slow component is associated with decreased efficiency of contractions and fatigue (31), the physiological significance of the HR slow component is less clear, and mostly anecdotal observations are present in the literature (17,30,32–36). A previous study from our group (11) demonstrated that the HR slow component i) occurs also during moderate-intensity exercise and ii) is more pronounced, percentage-wise, than the V̇O_2_ slow component during heavy-intensity exercise. Potential mechanisms responsible for the HR slow component include an increased body temperature (37) and an increased β-adrenergic drive (17). Independently from the cause, however, an increasing HR for the same work rate (HR slow component) would inevitably be associated with a decreasing work rate for the same HR, which was the phenomenon observed in the present and in our previous studies (11–13). In the present study, by observing a less pronounced work rate decrease for a constant HR in the patients of the BB group, we confirmed a potential role in the phenomenon attributable to β-adrenergic stimulation. However, considering the experimental design of the present study, we cannot exclude that inherent group differences influenced our results. Therefore, future studies adopting a crossover experimental design, to be performed in healthy subjects and/or in patients, could be helpful to strengthen the findings of the present study.

The interplay between the V̇O_2_ and HR slow components is an issue that needs to be better elucidated. In the present study, the work rate decrement was, in the no-BB group, more pronounced than that needed to prevent the V̇O_2_ slow component. Indeed, during HR_CLAMPED_ exercise in the no-BB group, V̇O_2_ actually decreased (by about 13%), confirming previous observations in healthy young subjects (11,12). Significant decreases were observed also for V̇CO_2_ and R, variables whose progressive increase would be indirectly associated with fatigue. Which mechanism(s) could be held responsible for the more pronounced decrease of work rate, compared with that necessary to keep V̇O_2_ and R constant (that is to say, to prevent slow components or continuous increases of these variables)? No answer to this question can derive from the present results. Another mechanism that might play a role in the determination of the HR slow component is the progressive decline in stroke volume associated with a parallel increase in HR, occurring after ~10 min of moderate-intensity exercise (38). It has been hypothesized that this phenomenon is linked to hyperthermia and dehydration-induced hypovolemia caused by prolonged exercise (37). Unfortunately, body temperature or indices of dehydration were not measured in the present study. However, the mirror image of the HR slow component occurred well before the 10th minute of exercise. Furthermore, Zuccarelli et al. (11) reported that, during both moderate- and heavy-intensity constant work rate exercises, there were no changes in stroke volume despite a progressive increase in HR. It seems unlikely that hyperthermia or dehydration occurred during the relatively short and very submaximal exercise bouts adopted in the present study.

The results of the present study should be relevant also in terms of aerobic exercise prescription. Training intensity is often prescribed at fixed HR value (4,6–10), mainly for its ease of use, and this common practice is based on the concept of a linear relationship between HR, V̇O_2_, and work rate (4). However, previous studies by our group (11–13) and the present one clearly show that this notion does not hold true. Exercise prescription at fixed HR values, slightly higher than that corresponding to GET (as it is often done when aerobic training is involved), is inevitably associated, even within a relatively short time period (about 15 min), with progressive and significant work rate and V̇O_2_ decreases. This phenomenon occurred in young healthy subjects (11,12), in obese adolescents (13) and in cardiac patients (no-BB patients of the present study). Exercise prescription at a fixed HR value, therefore, may not allow to adequately control the metabolic stimulus and presumably the adaptations to training.

Exercise intensity prescription based on fixed HR values is widely utilized also among patients with cardiovascular diseases (see, e.g., Refs. [10,15]). In the present study, we demonstrate that, also in this population, even a short duration task (15 min), which corresponds to half of the minimum duration of aerobic exercise recommended by guidelines (10), leads to a substantial reduction in work rate (−27%) and in V̇O_2_ (−13%). In the no-BB group, the work rate progressively decreased from a value slightly above GET (high-intensity domain [39]) at the beginning of the HR_CLAMPED_ exercise to a value corresponding to GET (boundary between high-intensity and moderate-intensity domains [39]) at the end of 15-min task (Fig. 3). During exercise training sessions of longer duration (e.g., 30–60 min, which is the typical duration prescribed by guidelines [4,10]), a shift to the moderate-intensity domain seems likely, possibly altering the exercise training stimulus. In more general terms, the effects on training efficacy deriving from keeping HR constant or work rate constant, during training sessions, remain to be specifically evaluated in future studies. The aforementioned issue seems to be attenuated in BB, in whom the work rate decrease during HR_CLAMPED_ was less pronounced than in no-BB, and presented a plateau after about 9 min of exercise. In BB patients, moreover, no V̇O_2_ decrease was observed during HR_CLAMPED_.

## CONCLUSIONS

In conclusion, in coronary artery disease patients in stable conditions, during a 15-min exercise on a cycle ergometer initially set at a work rate corresponding to an HR slightly above GET (as frequently done for aerobic exercise prescription), to keep HR constant, work rate substantially decreased. Confirming our hypothesis and suggesting a potential role by β-adrenergic stimulation, the work rate decrease was less pronounced (about 16%) in the patients treated with BB versus that observed (about 27%) in patients not treated with BB (no-BB). The decrease in work rate at a fixed HR may represent, also in cardiac patients, a sign of impaired exercise tolerance (lower work rate for the same HR) and makes aerobic exercise prescription based on fixed submaximal HR values rather problematic. The issue is less relevant in patients treated with BB. Future studies will have to define if training intensity should be prescribed at a fixed HR or at a fixed work rate.
